# Endoscope-Assisted Evacuation of an Acute Subdural Hematoma in an Elderly Patient With Refractory Nonconvulsive Status Epilepticus: An Illustrative Case

**DOI:** 10.7759/cureus.63817

**Published:** 2024-07-04

**Authors:** Mika Arai, Kenta Nakase, Ryota Sasaki, Fumihiko Nishimura, Ichiro Nakagawa

**Affiliations:** 1 Neurosurgery, Nara Medical University, Kashihara, JPN

**Keywords:** endoscopic assisted key hole surgery, post-traumatic seizures, elderly, non-convulsive status epilepticus, acute subdural hematomas

## Abstract

Traumatic brain injuries lead to post-traumatic seizures (PTS), with acute subdural hematomas (ASDH) posing a particularly elevated risk. The development of refractory nonconvulsive status epilepticus (NCSE) in such cases, especially in older patients, requires immediate and effective management. This case report highlights the improvement of refractory NCSE in an elderly patient with ASDH through endoscope-assisted evacuation. An 88-year-old woman was hospitalized for dysarthria and right hemiparesis 3 days after a fall. Computed tomography (CT) revealed a left hemispheric ASDH, 9 mm thick, along with minor traumatic subarachnoid bleeding in the interpeduncular cistern. The initial treatment was conservative, including the administration of lacosamide at 100 mg/day. However, her consciousness deteriorated 4 days after admission, and she experienced convulsions in the right face and arm on day 5. Although the convulsions stopped after the administration of diazepam 10 mg IV and her consciousness temporarily improved, it worsened again on day 6, leading to a diagnosis of NCSE on an electroencephalogram (EEG). Despite aggressive pharmacological interventions with fosphenytoin (750 mg initially followed by 262 mg/day) and phenobarbital (625 mg/day), the patient’s cognitive state and EEG findings did not improve. Consequently, on the 13th day, she underwent an endoscopic procedure to remove the SDH, which alleviated her symptoms and ended the seizures. This case demonstrates that even the absence of a significant mass effect from ASDH can trigger NCSE, underscoring the necessity for swift diagnosis and consideration of surgical options when conventional treatment fails. Endoscope-assisted evacuation is a safe and effective treatment option, particularly in older patients.

## Introduction

Acute subdural hematomas (ASDH) are commonly observed in younger individuals. However, this condition often occurs in the elderly and tends to result in poor clinical outcomes [1]. In elderly patients, low-velocity falls within the home environment are the primary contributors to these injuries [2]. The incidence of ASDH has been increasing, a trend partially attributable to the increasing number of individuals undergoing more comprehensive antithrombotic and anticoagulant therapies and also due to the aging population. [3,4]. Mortality following ASDH varies based on several factors, including patient age, Glasgow Coma Scale (GCS) score, extent of hemorrhage, and duration between the ASDH event and treatment initiation [4]. Post-traumatic seizures (PTS) are also independent markers of poor functional and social outcomes [4]. In elderly patients, the indications for surgery must be carefully determined because of low surgical tolerance. However, some patients have refractory non-convulsive status epilepticus (NCSE), and medication alone cannot resolve this situation. Here, we present an illustrative case of refractory NCSE that improved after endoscope-assisted evacuation of ASDH.

## Case presentation

**Figure 1:**
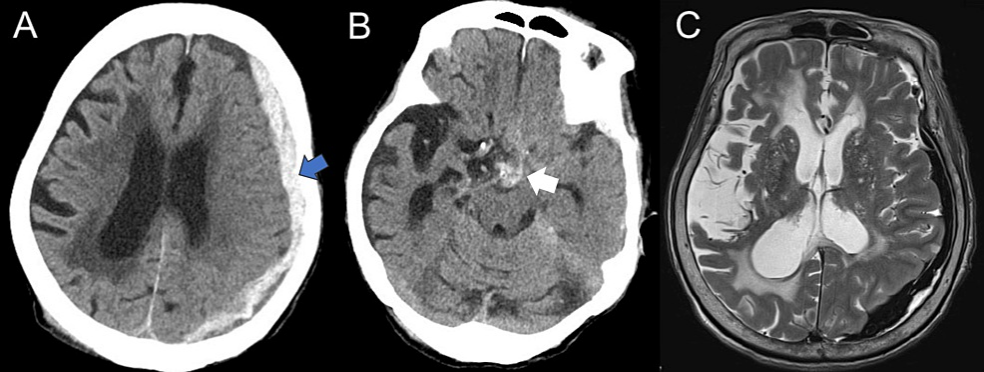
Initial computed tomography imaging and magnetic resonance imaging (A,B) Initial computed tomography (CT) images showing left hemispheric acute subdural hematoma (ASDH) (blue arrow) and mild traumatic subarachnoid hemorrhage in the interpeduncular cistern (white arrow). Magnetic resonance imaging (MRI) showing the same lesions, with hypointensity in the axial T2-weighted image (C). Additionally, MRI and CT showed hydrocephalus ex vacuo and the right predominant periventricular cerebrospinal fluid (CSF) leaks. No contusion or diffuse axis injuries were observed.

**Figure 2:**
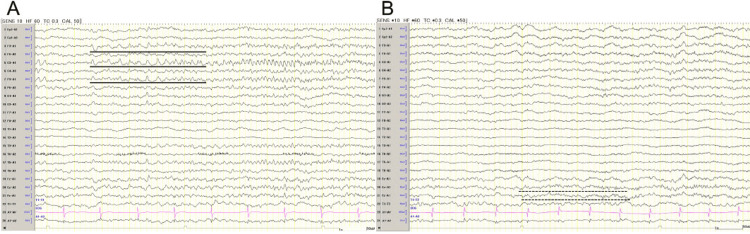
Electroencephalogram (EEG) findings (A) EEG on day 4 shows repetitive ictal patterns arising from the left fronto-parietal area (black line). (B) EEG on day 11 shows repetitive ictal patterns arising from the midline area (dotted line).

An 88-year-old woman weighing 33 kg presented with progressive dysarthria and right hemiparesis three days after a fall. On admission, she presented with a GCS score of E4V3M5, dysarthria, and right hemiparesis (MMT 4/5). Cranial CT scan revealed a left hemispheric ASDH, 9 mm thick, without a midline shift. However, it showed a mild traumatic subarachnoid hemorrhage in the interpeduncular cistern, hydrocephalus ex vacuo and the right predominant periventricular cerebrospinal fluid (CSF) leaks (Figure 1A, 1B). We preferred conservative therapy first because it did not fulfill the following surgical indications: ASDH less than 10 mm in thickness and mild consciousness disturbance. A nonconvulsive seizure was suspected, and lacosamide 100 mg/day was started on the same day. Although the CT scan showed no progression of ASDH, her consciousness deteriorated 4 days after admission (GCS E1V3M5). MRI revealed no other lesions such as contusions or diffuse axonal injury (Figure 1C). Laboratory data showed no evidence of electrolyte abnormalities, glucose metabolism abnormalities, or dehydration. Five days after admission, she experienced convulsions in the right face and arm. Although the convulsions stopped after the administration of diazepam 10 mg IV and her consciousness temporarily improved (GCS E3V4M5), it worsened again 6 days after admission (GCS E1V3M5). EEG revealed an ictal pattern in the left frontoparietal region, leading to a diagnosis of NCSE (Figure 2A). Subsequently, despite the administration of fosphenytoin sodium hydrate (750 mg on the first day and 262 mg/day on the second day) infused via IV for 30 minutes 7-8 days after admission and phenobarbital 625 mg/day infused via IV over 10 minutes 11 days after admission for NCSE, the persistent disturbance of consciousness and EEG findings remained unimproved (Figure 2B). Therefore, considering the need for subdural hematoma drainage, the patient underwent endoscope-assisted evacuation for ASDH 13 days after admission.

Surgical procedure

**Figure 3:**
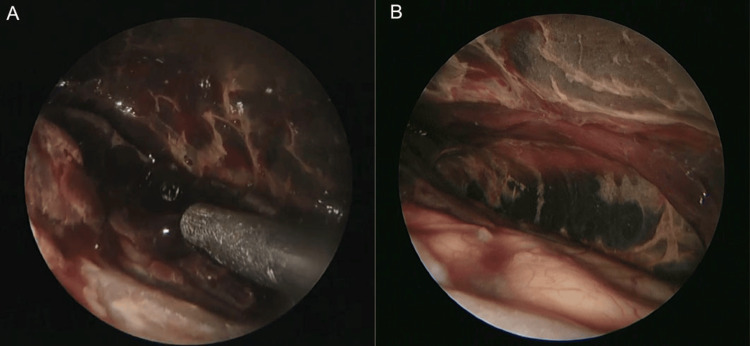
Intraoperative findings (A) A solid clot located in the subdural space is removed via a suction tube. (B) Subdural space after hematoma evacuation.

The patient underwent endoscope-assisted evacuation of ASDH under general anesthesia. We selected the site for craniotomy at the thickest part of the hematoma using neuronavigation. Then, a straight skin incision of 5 cm was made, and a 4-cm-diameter small parietal craniotomy was performed. The hematoma was jelly-like and had partially formed a capsule. The hematoma was aspirated with a suction tube while being observed with a 2.7-mm 0° and 30° rigid endoscope (Figure 3A, 3B). The rigid endoscope was held in the left hand, the suction cannula was held in the right hand, and the suction cannula was exchanged for bipolar forceps, as needed. The hematoma was removed using a suction cannula under the guidance of a rigid endoscope. When bleeding from a vessel on the brain surface was observed, a suction cannula was placed at the bleeding point, and coagulation was performed using monopolar electrocoagulation with a suction cannula. The bridging vein, which we identified as the source of bleeding, was coagulated using bipolar forceps. It was covered with absorbable Fibrillar™ hemostat (Surgicel®; Ethicon, Tokyo, Japan) for bleeding points. Finally, the subdural space was irrigated with artificial cerebrospinal fluid(Artcereb, Otsuka Pharmaceutical Factory, Inc., Tokushima, Japan). The dura mater was tightly closed and the bone was repositioned and stabilized using a fixture device, followed by closure of the scalp.

Postoperative course

**Figure 4:**
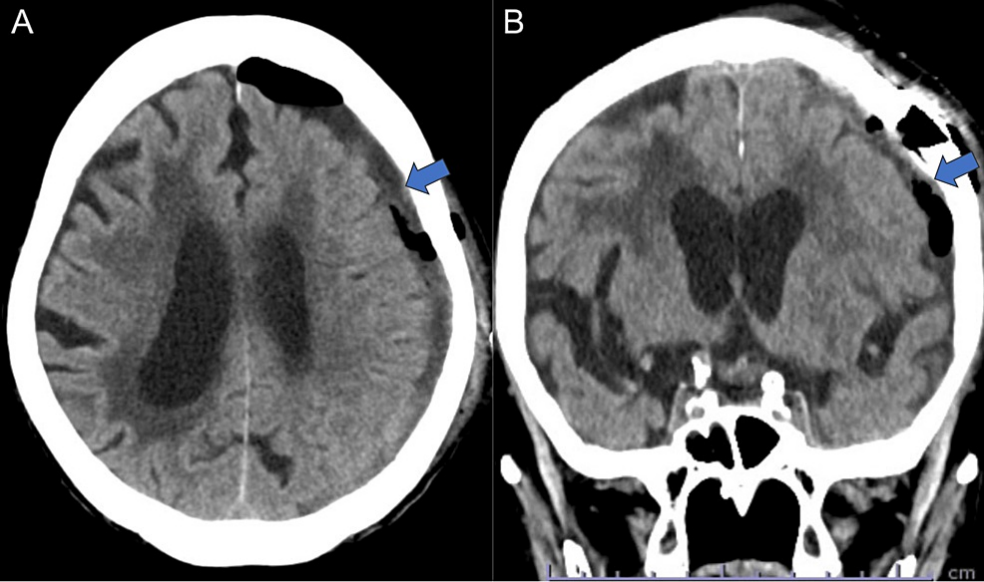
Postoperative computed tomography imaging Axial computed tomography (CT) images 1 day after endoscopic surgery showing gross total hematoma evacuation (blue arrows).

**Figure 5:**
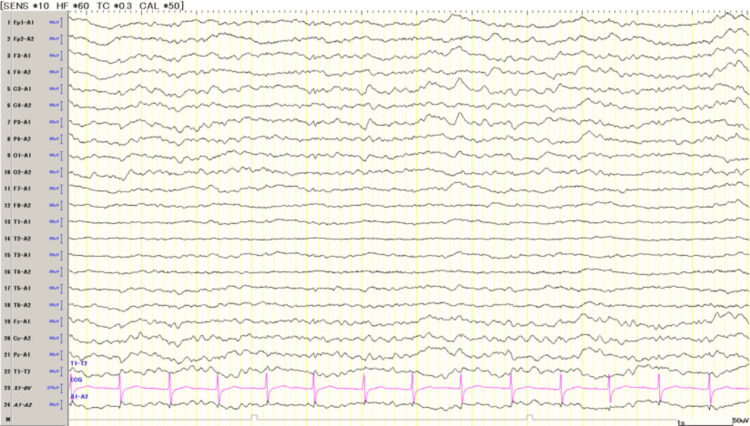
Electroencephalogram after surgery Electroencephalogram after surgery shows only intermittent slow waves in the left frontal area without any epileptiform discharges.

Postoperatively, the postoperative course was uneventful, and the patient showed improved consciousness (GCS E4V4M6) with no neurological deficits. A CT scan performed one day after the surgery showed near-total hematoma evacuation (Figure 4). EEG taken 15 days after admission showed that NCSE was completely suppressed (Figure 5). At discharge, on postoperative day 34, the patient’s modified Rankin Scale score was 4 due to disuse atrophy.

## Discussion

In elderly patients, ASDH is frequently associated with low-energy impacts resulting from falls at ground level [5,6]. However, Trevisi et al. highlighted a significant incidence of adverse outcomes in patients aged ≥ 70 years who were admitted for ASDH. Specifically, 31% of the patients died during their hospital stay, while an additional 32% were discharged either in a vegetative state (15%) or with a severe disability (17%). Only 22% of patients returned home upon discharge [6]. The surgical intervention approach for elderly patients with ASDH lacks standardization primarily because older patients frequently have comorbidities that constrain the options for surgical and anesthetic management. In cases where patients exhibit more favorable neurological conditions, a careful waiting strategy for hematoma chronicization is occasionally used [7,8].

However, 24%-36% of ASDH cases are compromised by PTS, and the occurrence of PTS is an independent marker of poor functional and social outcomes [9-11]. Swisher et al. performed continuous electroencephalogram monitoring in 243 ICU patients and reported a high seizure frequency of 43% in a subgroup with ASDH [12]. Additionally, although severe ASDH with intraparenchymal injury can cause PTS, some PTS also occurs in patients with fewer mass effects of ASDH. It is hypothesized that hematoma irritates the cortical surface with blood molecules, and later with its degradation products, as a pathophysiological mechanism for the high epileptogenicity of ASDH. Iron released from extravasated hemoglobin can trigger ROS generation of reactive oxygen species [13,14].

Medication is the first-line treatment for PTS. Commonly used antiepileptic drugs (AEDs) include levetiracetam, phenytoin, and valproic acid, which are selected based on their efficacy, side effect profiles, and patient-specific factors. Despite the use of these medications, some patients may develop status epilepticus, a severe and prolonged seizure state that occurs in approximately 0.5%-0.7% of cases. Furthermore, approximately 30% of patients with status epilepticus may progress to refractory status epilepticus, where seizures do not respond to standard treatments and require more aggressive management strategies such as continuous intravenous infusions of AEDs or anesthetics [15,16].

Surgery is recommended in patients with symptomatic ASDH greater than 10 mm thick and/or with an associated midline shift greater than 5 mm, a decreasing Glasgow Coma Scale score, or signs of herniation [15]. In elderly patients, the indications for surgery must be carefully determined because of the low surgical tolerance. However, some patients have refractory NCSE and medication alone cannot resolve this situation.

Therefore, it is necessary to select alternative treatment options for patients with refractory disease. Some studies have reported successful endoscope-assisted evacuation as a minimally invasive procedure in elderly patients with ASDH [17,18]. Compared with conventional surgery, endoscope-assisted evacuation with a small craniotomy may have the following benefits: shorter operative time, less intraoperative blood loss, and small skin incision and craniotomy [17,18]. These benefits may help to prevent the accumulation of epidural hematomas and lower the risk of infection after the procedure. Moreover, local anesthesia can be considered for patients in whom general anesthesia presents a high risk of comorbidities [19]. 

However, there are several disadvantages: the working space is narrow, surgical access is limited, and endoscope-assisted evacuation cannot decompress the brain swelling. Intraparenchymal contusions and hematoma (IPHs) progression are inherent to the injury process and can increase brain edema following trauma. Therefore, endoscope-assisted evacuation is not suitable for patients with ASDH or significant IPHs. Miki et al. showed that brain swelling, large IPHs, fractures potentially accompanied by dural and/or sinus injuries, and severe coagulopathy are contraindications for endoscope-assisted evacuation in elderly patients with traumatic ASDH. They also concluded that patients with greater time elapsed after injury (> 24 h), no large IPHs, and no brain swelling were more likely to be safer candidates for endoscope-assisted evacuation [19]. 

In a recent review, mortality rates ranged between 0% and 40%, whereas favorable outcomes ranged between 26.7% and 96.4% for ASDH in elderly patients undergoing endoscopic-assisted surgery. Re-bleeding was the most commonly reported complication, occurring in 0-13% of cases [20]. Although the patient in our case had improved symptoms and no complications, these findings suggest that although endoscope-assisted evacuation can offer significant benefits in terms of reduced mortality and improved outcomes, it is not without risks. The variability in outcomes highlights the importance of careful patient selection and the need for experienced surgical teams to minimize complications. Moreover, the potential for rebleeding underscores the necessity for rigorous postoperative monitoring and timely intervention if complications arise. Further research is needed to standardize protocols and optimize techniques to enhance the safety and efficacy of this minimally invasive approach for elderly patients with ASDH.

## Conclusions

Hematoma itself can cause NCSE even in the absence of a significant mass effect from ASDH. Therefore, it is essential to immediately detect and consider surgery for medication-resistant cases. Endoscope-assisted surgery may be an appropriate treatment option for ASDH with refractory NCSE in elderly patients. It is crucial to monitor additional cases and evaluate the long-term outcomes to establish the efficacy of this approach.
